# Supplementary material for: FlowMRI-Net: A generalizable self-supervised 4D flow MRI reconstruction network
Source: J Cardiovasc Magn Reson. 2025 May 16;27(2):101913. doi: 10.1016/j.jocmr.2025.101913 (PMC12271072; doi:10.1016/j.jocmr.2025.101913)

Supplementary material of “FlowMRI-Net: A Generalizable Self-Supervised 4D Flow MRI Reconstruction Network”

Luuk Jacobs^1^, Marco Piccirelli^2^, Valery Vishnevskiy^1^, Sebastian Kozerke^1^

^1^ Institute for Biomedical Engineering, University and ETH Zurich, Zurich, Switzerland

^2^ Department of Neuroradiology, University Hospital Zurich, Zurich, Switzerland

# Prospective undersampling

Aortic 4D flow data was acquired continuously for approximately one hour per volunteer using a pseudo-spiral (or variable-density radial, VDrad) sampling pattern. One cardiac bin is defined as a user-defined number of readouts forming one arm (a), the next cardiac bins is then sampled upon rotation of the arm by the Golden angle (b). Note that the k-space centre is sampled for each arm. After a continuous scan, a single cardiac bin is filled with many arms, forming a pseudo-random variable density pattern (c), where the centre of k-space is sampled more than the periphery (d, clipped at 20 counts). With retrospective respiratory binning into three bins, only a third of the readouts are used, still resulting in a pseudo-random variable density pattern (e), where the centre of k-space is sampled more than the periphery (f, clipped at 10 counts).

1. b) c) d) e) f)


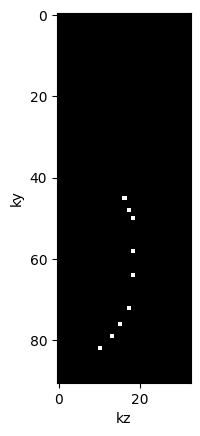

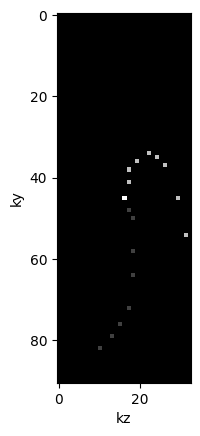

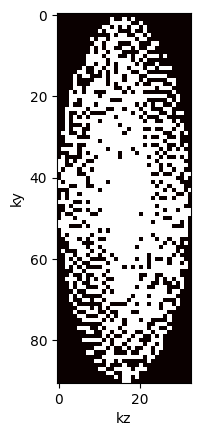

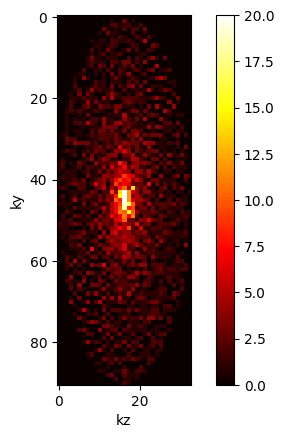

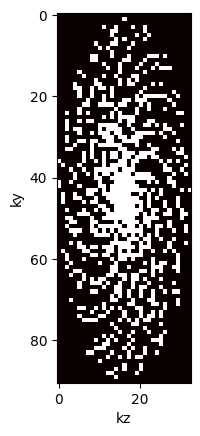

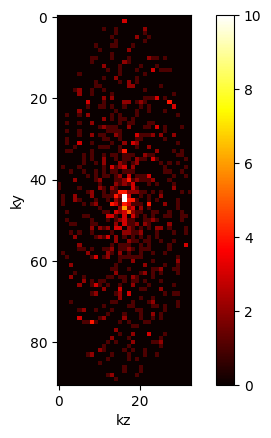


Note that VDrad sampling facilitates prospective undersampling by simply leaving out the last number of readouts, depending on the desired undersampling factor, without sacrificing k-space coverage.

# CS-LLR hyperparameters

A CS-LLR reconstruction was performed on the densely-sampled ($R=4$) aortic scans, which were considered to be 3D ground truths. An optimal value of $\lambda_{LLR}$ was empirically determined per subject by minimizing noise during diastolic phase without increasing the underestimation of peak velocity in FH direction compared to the fully-sampled 2D ground truth acquired during breath-holding. The resulting 3D ground truth of the validation set was used for automatic hyperparameter optimization of the baseline CS-LLR reconstructions of higher undersampling rates. The CS-LLR reconstructions were fixed to 70 iterations and a patch size of 16x16x16, where only $\lambda_{LLR}$ was optimized per undersampling rate to minimize the velocity magnitude MSE inside the thoracic aorta with respect to the 3D ground truth (see Fig. 1).


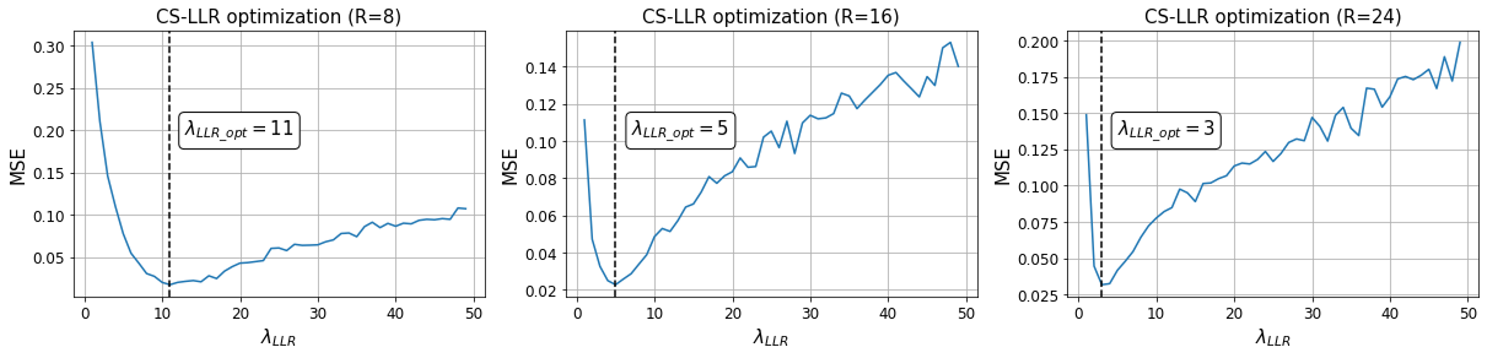


Figure 1: **Automatic grid search optimization for** $\boldsymbol{\lambda}_{\boldsymbol{LLR}}$ **in CS-LLR reconstructions of aortic data at** $\boldsymbol{R=8,16,24}$**.** The search spaces per optimization were limited to integers between 0 and 50, taking less than an hour each.

These optimized parameters were found to generalize poorly to cerebrovascular reconstructions for respective undersampling factors. Because no high-quality ground truth was available for automatic optimization of $\lambda_{LLR}$, the value was empirically optimized to best match the velocity curves of the R=2 GRAPPA reconstruction in the test set, resulting in $\lambda_{LLR}=1, 0.5, 0.25$ for $R=8, 16, 24$, respectively. This “inverse crime” approach facilitated the comparison of FlowMRI-Net to the upper limit of CS-LLR, which in practice may be unachievable due to the lack of an R=2 GRAPPA reconstruction.

# Aortic peak velocity curves

A comparison of the peak velocity curves of the fully sampled 2D breath-hold reference (2D REF) and the 4D flow reconstructions (CS-LLR and FlowMRI-Net) for two undersampling factors (R=8,16) and three encoding directions (FH, RL, and AP), as also shown in Fig. 4 in the main text for one volunteer, are shown here for the remaining four volunteers in the test set:


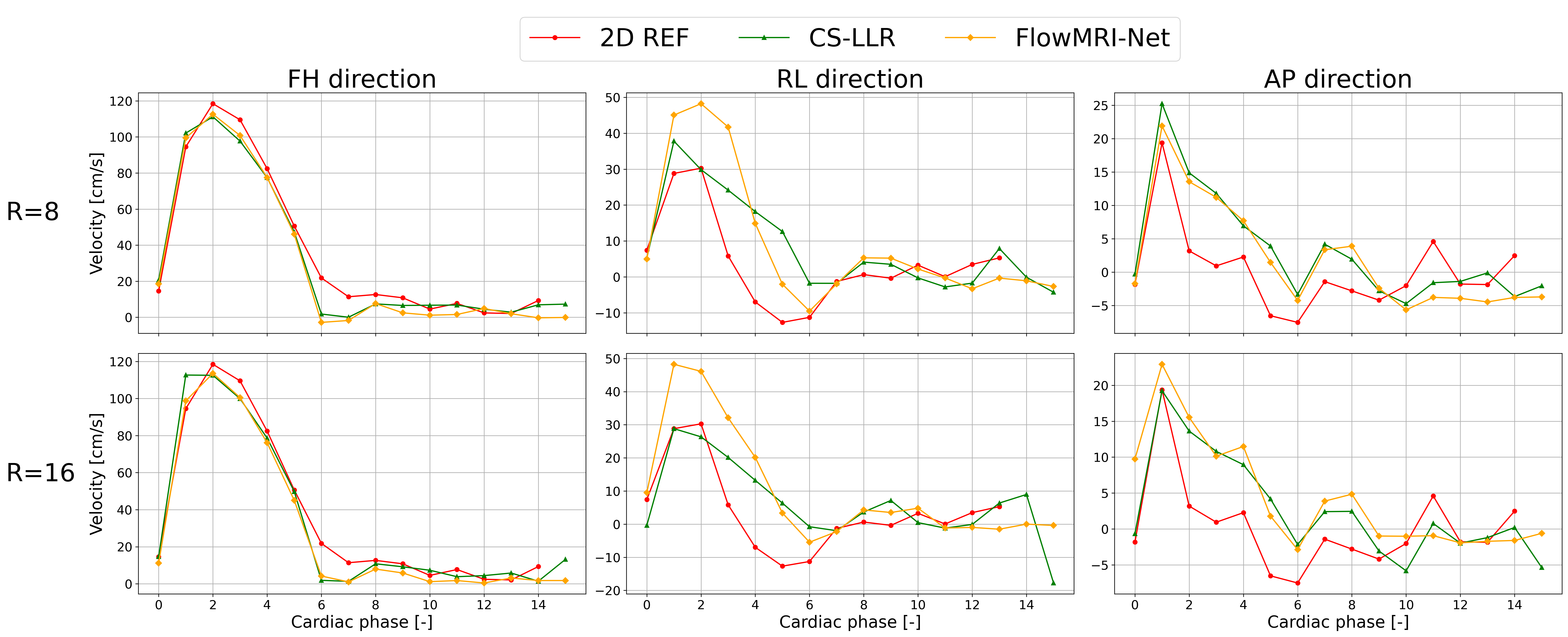


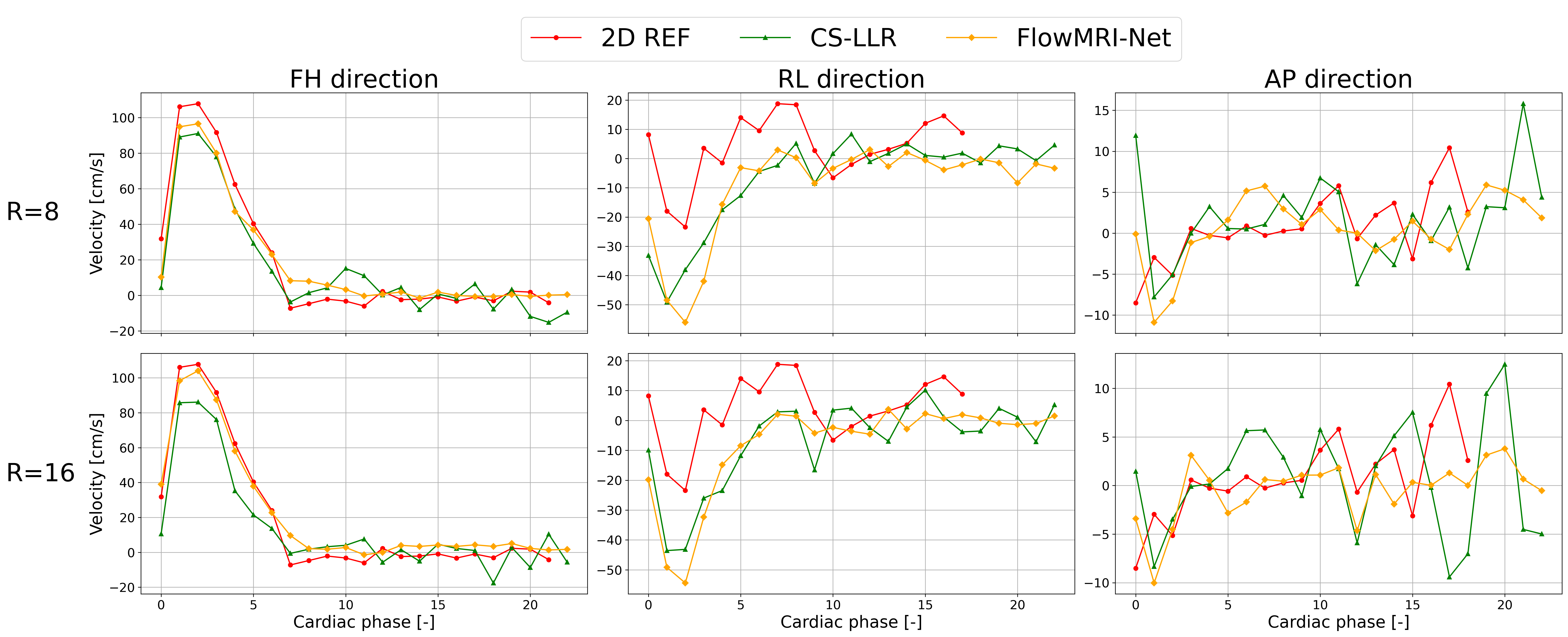


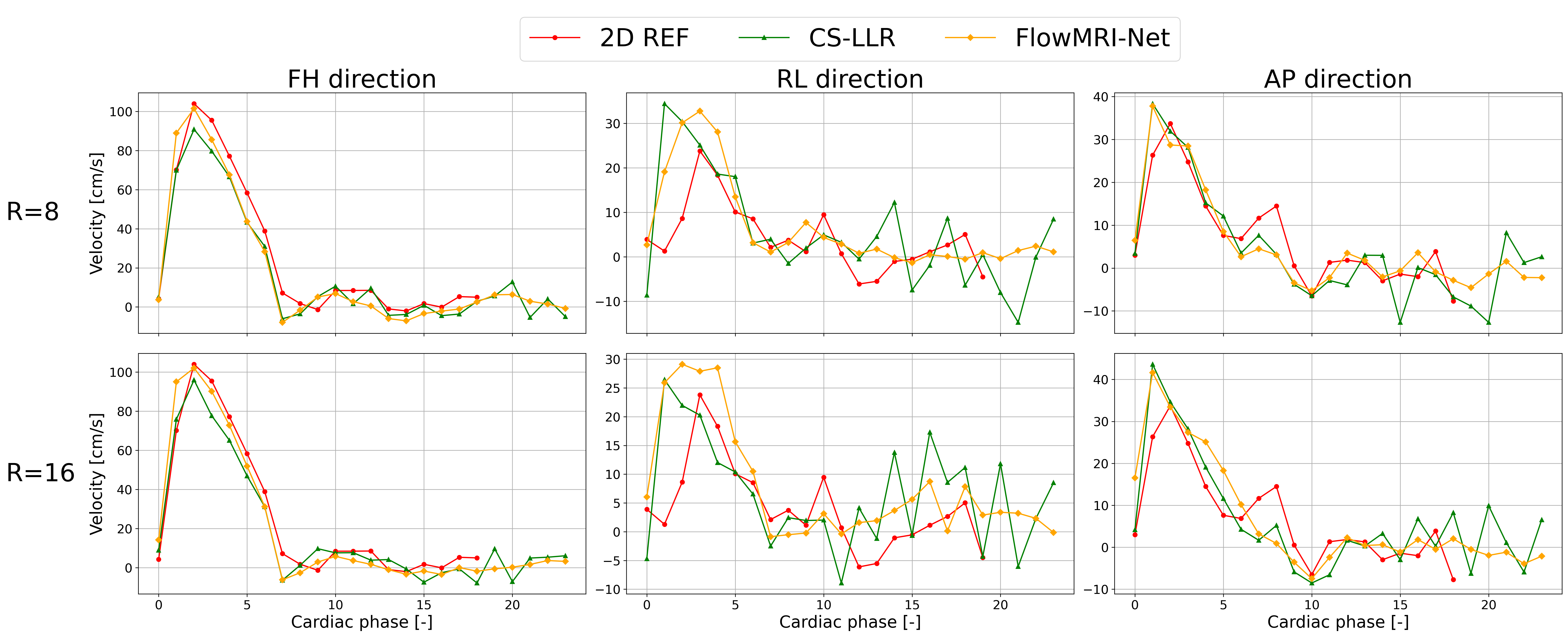


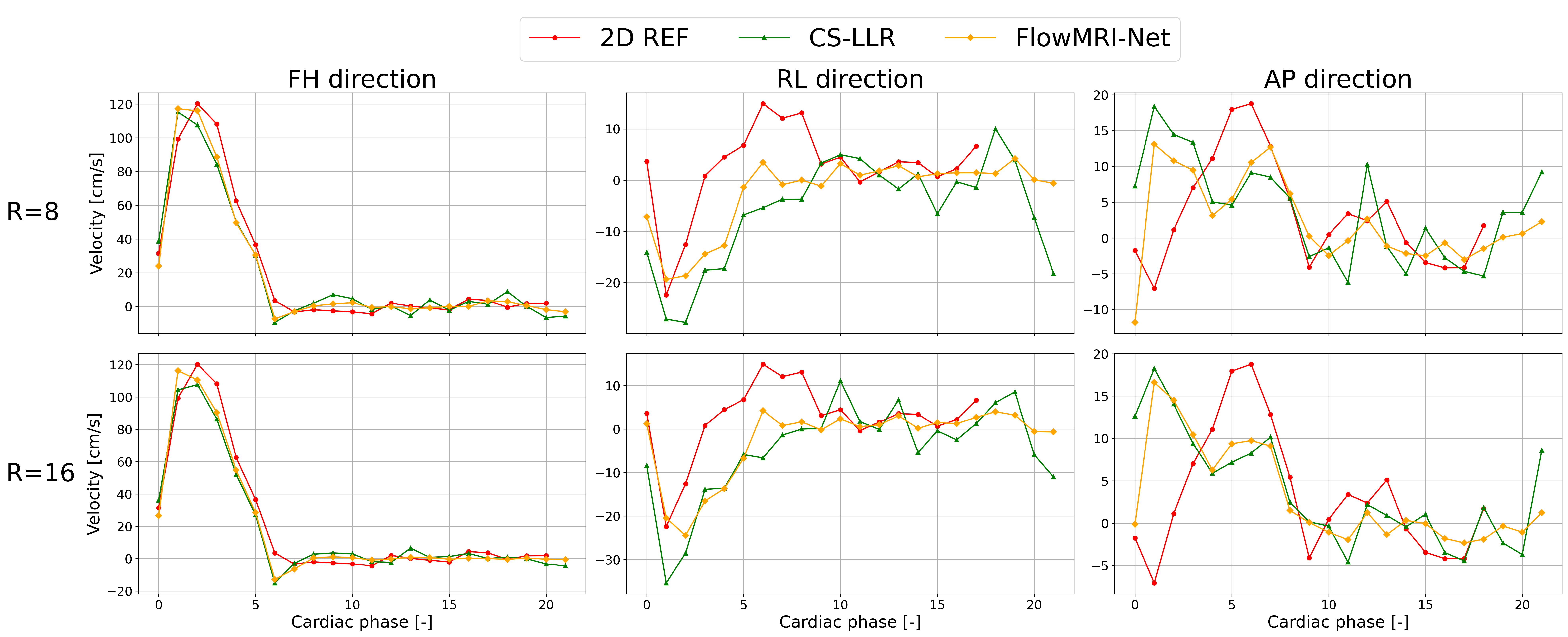

Supplement: Supplementary material [file mmc1.docx]
